# Tailored Thermoresponsive Polyurethane Hydrogels: Structure–Property Relationships for Injectable Biomedical Applications

**DOI:** 10.3390/polym17172350

**Published:** 2025-08-29

**Authors:** Miriam Di Martino, Lucia Sessa, Federica Romano, Stefano Piotto, Simona Concilio

**Affiliations:** 1 Department of Pharmacy, University of Salerno, Via Giovanni Paolo II, 132, 84084 Fisciano, SA, Italy; midimartino@unisa.it (M.D.M.); romano.federica999@gmail.com (F.R.); piotto@unisa.it (S.P.); 2 Bionam Center for Biomaterials, University of Salerno, Via Giovanni Paolo II, 132, 84084 Fisciano, SA, Italy

**Keywords:** thermoresponsive hydrogels, polyurethane-based networks, sol–gel transition, monomer composition tuning

## Abstract

Thermoresponsive hydrogels that undergo reversible sol-gel transitions near physiological temperatures are highly attractive for biomedical applications, such as injectable drug delivery and embolization therapies. In this study, a library of polyurethane-based hydrogels was synthesized via step-growth polymerization using polyethylene glycol (PEG) of varying molecular weights, different diisocyanates, and a series of functional diols derived from diethanolamine with increasing hydrophobicity. The resulting polymers exhibited sol–gel transition behaviors without the need for external crosslinkers, relying solely on non-covalent interactions. The thermal responsiveness was systematically investigated using UV–Vis turbidimetry, and the cloud point temperature (T_CP_) was found to be tunable within a range of 26–49 °C by modulating the monomer composition. Statistical modeling identified PEG molecular weight and diol structure as the primary determinants of T_CP_, while diisocyanate type and diol-to-PEG ratio had negligible effects. Only diethanolamine (DEA)-based polymers formed stable hydrogels above a critical gelation temperature (LCGT), attributed to enhanced intermolecular interactions via free amine groups. In vitro degradation assays confirmed good hydrolytic stability under physiological conditions over four weeks, with degradation profiles strongly influenced by the PEG chain length and hydrophobic content. These findings establish a structure–property framework for the rational design of injectable, thermoresponsive polyurethane hydrogels with tailored sol–gel behavior for biomedical applications.

## 1. Introduction

Stimuli-responsive hydrogels, which undergo structural transitions in response to external or internal stimuli, such as temperature [1,2], pH [3,4], or light [5,6,7], have attracted considerable interest for applications in biomedical fields, such as controlled drug delivery, biosensing diagnostics, and tissue engineering [8,9,10,11,12]. Among them, thermoresponsive hydrogels can exhibit a sol-gel phase transition at a defined temperature, making them particularly promising as injectable biomaterials. Below this temperature, defined as the lower critical solution temperature (LCST), they remain in a liquid state, enabling minimally invasive administration. Upon reaching the LCST, they transition into a gel, forming a stable three-dimensional network [13,14,15].

Throughout this work, we explicitly distinguish the cloud-point temperature (T_CP_), quantified by UV–Vis turbidimetry from the lower critical gelation temperature (LCGT) that marks network percolation, as these two design parameters can differ and jointly govern in situ performance [16,17,18].

The LCST is a key design parameter, as it dictates the hydrogel’s responsiveness to thermal stimuli and its behavior in physiological conditions. Positioning T_CP_/LCGT just above room temperature and close to 37 °C enables syringeability during administration while ensuring rapid, on-demand solidification at the target site [16,17].

Polyurethanes (PUs) have emerged as a versatile class of synthetic polymers for hydrogel development, owing to their flexibility, biocompatibility, and tunable properties. Their segmented structure, comprising alternating hydrophilic (soft) and hydrophobic (hard) domains, provides precise control over mechanical and thermal characteristics. The soft, hydrophilic segments, typically derived from polyols, impart flexibility and elasticity to the material by allowing chain mobility. In contrast, the hard, hydrophobic segments, formed by diisocyanates and short chain extenders, aggregate into rigid microdomains through hydrogen bonding and van der Waals interactions. These domains act as physical crosslinks that enhance tensile strength, modulus, and thermal stability. The degree of microphase separation and the size of the hard domains thus critically influence the overall mechanical performance and thermal behavior of the polyurethane [19,20,21,22].

In segmented PUs, microphase-separated hard domains serve as reversible physical junctions, while PEG-rich soft domains dictate solvophilicity and thermal dehydration; this interplay governs both thermogelling and mechanics [23,24].

Relative to widely used thermogelling benchmarks, PU hydrogels can address specific limitations of existing alternatives. PNIPAM-based hydrogels, despite sharp LCST behavior near 32 °C, are often non-biodegradable and mechanically weak in the highly swollen state, motivating copolymer or composite strategies [25,26]. Pluronic (F127) gels, while convenient and fast-gelling, typically show modest gel strength and are readily cleared in vivo, which limits depot longevity and embolic persistence [27]. From a translational standpoint, platforms that avoid chemical crosslinkers or photoinitiators reduce concerns about residual reagents and simplify clinical workflows [28].

While numerous studies have reported on the temperature sensitivity of polyurethane-based hydrogels [29,30,31,32], many systems still depend on crosslinking agents for gelation [30,33,34,35]. However, reliance on in situ chemical crosslinking can introduce additional cytocompatibility and handling constraints compared with purely physical gelation [28].

This work presents the synthesis and characterization of thermoresponsive polyurethane-based hydrogels featuring a segmented architecture, where polyethylene glycol (PEG) serves as the flexible, thermoresponsive component and diisocyanates and low-molecular-weight diols comprise the rigid and hydrophobic part. We systematically investigate how variations in PEG molecular weight, diisocyanate type, and functional diols derived from alkylated diethanolamine affect the materials’ thermal responsiveness and gelation behavior. By constructing a combinatorial library and applying statistical modeling, we identify PEG molecular weight and diol structure as the dominant predictors of T_CP_, whereas diisocyanate identity and diol-to-PEG ratio exert negligible influence. Notably, only diethanolamine (DEA)-derived polymers form stable hydrogels above a critical LCGT, consistent with enhanced intermolecular interactions imparted by secondary amines that modulate hard-domain packing and hydrogen-bond density [23,36].

By fine-tuning these molecular parameters, we developed hydrogels with customizable sol–gel transition profiles, making them strong candidates for injectable biomedical applications, including controlled drug release and embolization therapy [37,38,39,40,41,42]. Within the embolization landscape, where injectable liquids must solidify in situ and remain mechanically robust, recent hydrogel-based agents frequently rely on dual or covalent crosslinking to achieve persistence, underscoring the value of a crosslinker-free, physically gelled PU design [43,44,45].

A key advantage of the hydrogels developed in this study is their ability to undergo spontaneous gelation without the need for external crosslinkers, relying solely on non-covalent interactions. Furthermore, these hydrogels maintain their three-dimensional structure even below the gelation temperature, providing enhanced stability at the application site.

## 2. Materials and Methods

All reagents and solvents were purchased from Sigma-Aldrich (Milan, Italy) and Carlo Erba Reagents. PEG diols (PEG_400_, Mn = 400 Da; PEG_1000_, Mn = 1000 Da), Hexamethylene diisocyanate (HMDI), Isophorone diisocyanate (IPDI), Diethanolamine (DEA), *N*-methyldiethanolammine (Me-DEA), and Tin (II) ethyl hexanoate (Sn(Oct)_2_) were dried before use. The other reagents were used without further purification.

The optical transmittance of polyurethane solutions was determined using a UV–Vis Varian Cary 50 spectrophotometer equipped with a single-cell Peltier accessory. Measurements were performed at a wavelength of 600 nm, with the sample solutions (1.0 mg mL^−1^) prepared in phosphate-buffered saline (PBS, 150 mM, pH 7.4). The solutions were equilibrated for 10 min before each measurement. ^1^H nuclear magnetic resonance (^1^H NMR) spectra were recorded at room temperature with a DRX 400 spectrometer (Bruker, Billerica, MA, USA). Samples were dissolved in deuterated dimethyl sulfoxide (DMSO-d_6_). The infrared spectra were acquired using an FT-IR spectrometer (Spectrum Two FT-IR, PerkinElmer, Waltham, MA, USA) at a resolution of 2.0 cm^−1^, with 64 scans, across a wavelength range of 4000–500 cm^−1^.

Biodegradability tests were conducted using a thermostatic bath (JULABO GmbH, Seelbach, Germany) at 37 °C in a PBS solution (150 mM, pH 7.4) containing 20 wt% samples.

A set of 11 thermoresponsive copolymer formulations were selected for statistical analysis based on their solubility and measurable cloud point temperature (T_CP_). The formulation variables considered were PEG molecular weight (PEG M_w_): 400 or 1000 Da; diol structure: diethanolamine (DEA), *N*-methyldiethanolamine (Me-DEA), or 3-methylbutyldiethanolamine (MeBut-DEA); diisocyanate type: hexamethylene diisocyanate (HMDI) or isophorone diisocyanate (IPDI); and molar ratio Diol:PEG: 50:50 or 75:25.

### 2.1. Synthesis of Tertiary Amine Diols

#### 2.1.1. Synthesis of 2-[Hexyl-(2-hydroxyethyl) Amino] Ethanol (Hex-DEA)

Diethanolamine (0.50 g, 4.76 mmol) was dissolved in 8.5 mL of dry acetonitrile. Next, Na_2_CO_3_ (0.60 g, 5.68 mmol) and Br-hexane (0.73 mL, 5.18 mmol) were added to the mixture. The mixture was stirred and heated at reflux for 48 h under a nitrogen atmosphere. After this time, the mixture was cooled, Na_2_CO_3_ was filtered out, and the solvent was evaporated. The residue was dissolved in chloroform and washed with a brine solution. The organic phase was anhydrified, and the solvent was evaporated under reduced pressure, yielding yellow oil. The yield was 88%.

^1^H NMR (400 MHz; CDCl_3_) δ: 0.80–0.84 (3H, m), 1.17–1.26 (6H, m), 1.37–1.39 (2H, m), 2.43–2.47 (2H, t), 2.57–2.60 (4H, t), 3.53–3.56 (4H, t).

#### 2.1.2. Synthesis of 2-[3-Methyl-butyl-(2-hydroxyethyl) Amino] Ethanol (MeBut-DEA)

Diethanolamine (0.50 g, 4.76 mmol) was dissolved in 8.5 mL of dry acetonitrile. Next, Na_2_CO_3_ (0.60 g, 5.68 mmol) and 1-Br-3-methylbutane (0.62 mL, 5.18 mmol) were added. The mixture was stirred and heated to reflux for 48 h under a nitrogen atmosphere. After this period, the mixture was cooled, Na_2_CO_3_ was filtered out, and the solvent was evaporated. The residue was dissolved in chloroform and washed with a brine solution. The organic phase was dried over Na_2_SO_4_, and the solvent was evaporated under reduced pressure, yielding a clear oil product with a 50% yield.

^1^H NMR (400 MHz; CDCl_3_) δ: 0.82–0.84 (6H, d), 1.26–1.32 (2H, m), 1.43–1.55 (1H, m), 2.46–2.49 (2H, t), 2.57–2.60 (4H, t), 3.54–3.56 (4H, t).

### 2.2. Synthesis of Polyurethanes

The copolymers were synthesized by reacting hexamethylene diisocyanate (HMDI) or isophorone diisocyanate (IPDI) with polyethylene glycol (PEG, molecular weight (Mn) = 400 or 1000) and a functional diol. In a typical polymerization, diols (2.25 mmol) and diisocyanate (2.25 mmol) were dissolved in dichloromethane. Sn(Oct)_2_ (5 mol%) was the catalyst. The polymerizations were carried out at 30 °C for 72 h. The resulting polymers were purified by precipitation in diethyl ether; the precipitates were filtered and dried under vacuum.

### 2.3. Hydrogel Preparation

A specific amount of polymers obtained was dissolved in a PBS solution (150 mM, pH 7.4) to achieve a final concentration of 20 wt%. The solutions were stored at 4 °C overnight until the polymer was fully dissolved. Then, they were heated from 4 to 60 °C in 2 °C intervals. The sol (flow)–gel (non-flow) phase transition temperature of the polymers in the buffer solution was recorded using the tube-inverting method.

### 2.4. In Vitro Degradation Tests

The samples at a 20 wt% concentration were incubated in phosphate-buffered saline (PBS, pH 7.4) at 37 °C, and their dry weight was recorded at specific time intervals to evaluate the extent of degradation under simulated physiological conditions. The variation in the dry weight of the samples was assessed at 7-day intervals over a 4-week period. At each interval, samples were removed from the solution, dried to a constant weight, and weighed.

The percentage of weight loss was calculated using the following equation:

(1) $$W_{l}\;(\%)=\frac{W_{o}\;-\;W_{t}}{W_{o}}\times 100$$

where *W_l_* is the weight loss, *W_o_* is the initial dry weight of the samples, and *W_t_* is the weight of the dried samples at a given time point *t*. All data are reported as mean ± standard deviation (SD) from three independent replicates.

### 2.5. Statistical Analysis Workflow

Statistical analyses were conducted in Python (v3.11) using pandas, statsmodels, seaborn, and matplotlib. This study consisted of the following:

1. Manual entry of experimental data into a structured DataFrame containing the following columns: PEG M_w_, Diol, Diisocyanate, Diol:PEG Ratio, and T_CP_;
2. Application of an ordinary least squares (OLS) regression model with the formula
  (2) TCP=β0+β1·PEG Mw+β2·Diol+β3·Diisocyanate+β4·Ratio
  All variables were treated as categorical variables using reference (dummy) encoding. In this model, β_0_ represents the intercept corresponding to the reference group, while the β_i_ coefficients quantify the marginal effect of each categorical level relative to its respective reference category;
3. Execution of a type II ANOVA on the fitted model to assess the statistical significance of each factor;
4. Generation of predicted T_CP_ values using the regression model across all possible combinations of formulation parameters, creating a full prediction matrix;
5. Visualization of the marginal effects of PEG M_w_ and diol structure on the predicted T_CP_ using bar plots;
6. Extraction of the model equation from regression coefficients.

#### Modeling of Gelation Probability

To investigate the factors influencing gel formation, we modeled the probability of gelation using a penalized logistic regression approach. The binary response variable Gel was defined as 1 for gel-forming systems and 0 for non-gelling formulations. The following categorical predictors were considered: PEG Molecular Weight (400 or 1000), type of diol (DEA, Me-DEA, MeBut-DEA), type of diisocyanate (IPDI or HMDI), and molar ratio of hydroxyl-bearing reagents (50:50 or 75:25). All categorical predictors were encoded using one-hot encoding, omitting the reference category to avoid collinearity. Given the small sample size and the indication of perfect separation in classical logistic regression, we used an L_2_-regularized logistic regression model implemented in scikit-learn, which improves generalization and prevents divergence in coefficient estimation. The pipeline included preprocessing and model fitting:

Define X = categorical predictors (PEG_Mw, Diol, Diisocianate, Ratio);
Define y = binary outcome (Gel).
Apply one-hot encoding to X, dropping first level of each category.
Create a pipeline:
Preprocessing → LogisticRegression (L2 penalty, solver=lbfgs)
Fit the model to (X, y).
Predict gelation probabilities:
Prob_Gel = model.predict_proba(X)[:, 1]

The outcome of this model was the predicted probability of gelation for each formulation, reported in the next section.

## 3. Results and Discussion

**Figure 1:**
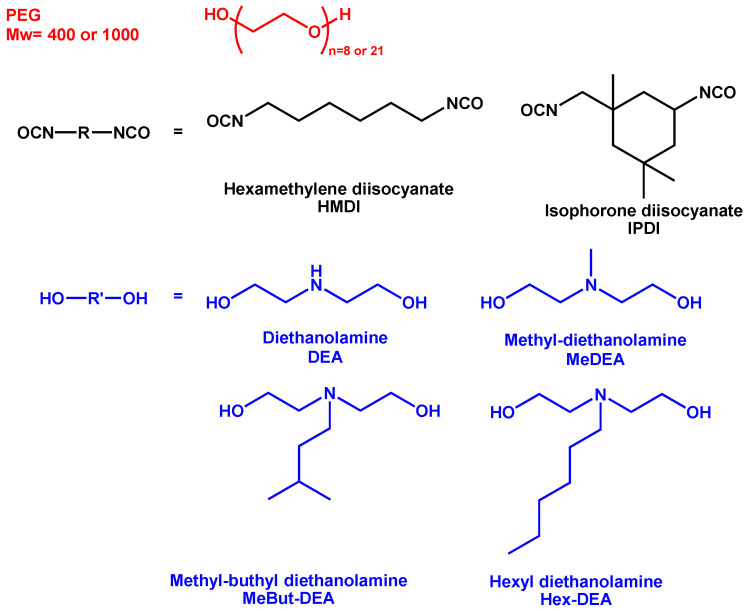
Chemical structures of the monomers used.

In this work, various monomers were used to synthesize polyurethane-based hydrogels, including polyethylene glycol (PEG) with different molecular weights, a range of diisocyanates, and functional monomers based on diethanolamine with varying substitutions. The primary objective was to evaluate the impact of monomer selection and composition on the final properties of the hydrogels, specifically examining how variations in these components influence the structural and thermoresponsive characteristics of the resulting materials. PEGs at two different molecular weights were used, PEG M_w_ 400 and 1000, along with two different diisocyanates (hexamethylene diisocyanate, HMDI, and isophorone diisocyanate, IPDI), diethanolamine (DEA), and three alkylated derivatives (Me-DEA, MeBut-DEA, Hex-DEA) as functional diols. Me-DEA has a methyl group, MeBut-DEA has a 3-methyl butyl group, and Hex-DEA has a hexyl group on the nitrogen atom of diethanolamine. Figure 1 reports the chemical structures of the monomers used.

PEG polyols were selected as flexible components to impart thermoresponsive properties to the resulting polymers. This thermoresponsiveness arises from the ability of PEG chains to reversibly hydrate and dehydrate in aqueous environments, leading to sol-gel transitions at specific temperatures. The gelation temperature of PEG-containing polymers can be tuned by adjusting the PEG chain length and concentration, as reported in [46,47,48].

Diethanolamine derivatives were selected to assess the impact of various substitutions and to enhance the system’s hydrophobic characteristics [30,49].

The two aliphatic diisocyanates employed, HMDI and IPDI, were selected to investigate the potential influence of hard-segment structure on the thermoresponsive behavior of the resulting polyurethanes. HMDI is a linear aliphatic diisocyanate that provides greater chain flexibility and lower steric hindrance, whereas IPDI contains a bulky cycloaliphatic ring, introducing higher rigidity and steric constraints that may affect microphase separation and chain packing. Both are widely used in biomedical polyurethane synthesis due to their lower cytotoxicity compared to aromatic diisocyanates [50,51].

### 3.1. Synthesis of Polyurethane Polymers

**Figure 2:**
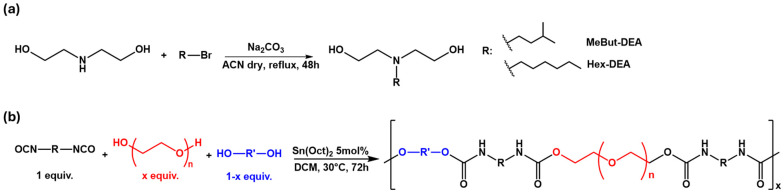
General schemes for the synthesis of (**a**) alkylated diethanolamine derivatives and (**b**) polyurethane polymers.

The alkylated diethanolamine derivatives were obtained by reacting different bromoalkyl compounds with diethanolamine in dry acetonitrile, with the addition of sodium carbonate [52] (Figure 2a).

Polyurethanes are obtained by one-pot condensation polymerization (Figure 2b) of monomers in different amounts in the presence of a Tin (II) catalyst [30].

Varying the monomers and their properties yielded fifteen different polyurethane polymers. Table 1 shows the relative amounts of reagents used to prepare different polyurethane polymers.

The polymerizations were monitored by FT-IR and ^1^H NMR spectroscopy.

### 3.2. Structural Characterizations of Polyurethane Polymers

**Figure 3:**
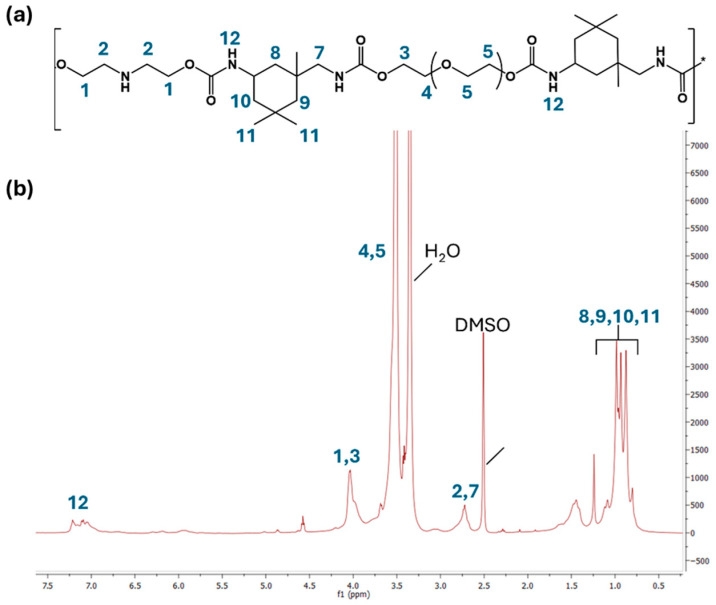
(**a**) Chemical structure of polyurethane polymer (sample C1) and (**b**) ^1^H NMR spectrum of the sample. In the molecule and spectrum, the protons are indicated.

^1^H NMR and IR spectroscopy were used to characterize all obtained polymers and confirm the polymerization process. The proton NMR spectrum (Figure 3b) of sample C1 shows some of the characteristic signals of the monomers, including the peaks between 0.8 and 1 ppm relating to the -CH_2_ and -CH_3_ of diisocyanate (numbers 8, 9, 10, and 11 in Figure 3a,b) and an intense peak at around 3.5 ppm relating to the -CH_2_ of PEG polyol. Especially the signals at 4.00 ppm corresponding to the -CH_2_ methylene protons (indicated by numbers 1 and 3 in Figure 3a,b) linked to the urethane groups and the signal at 7.00 ppm corresponding to the -NH amide proton (indicated by number 12 in Figure 3a,b) confirm the formation of polyurethane.

**Figure 4:**
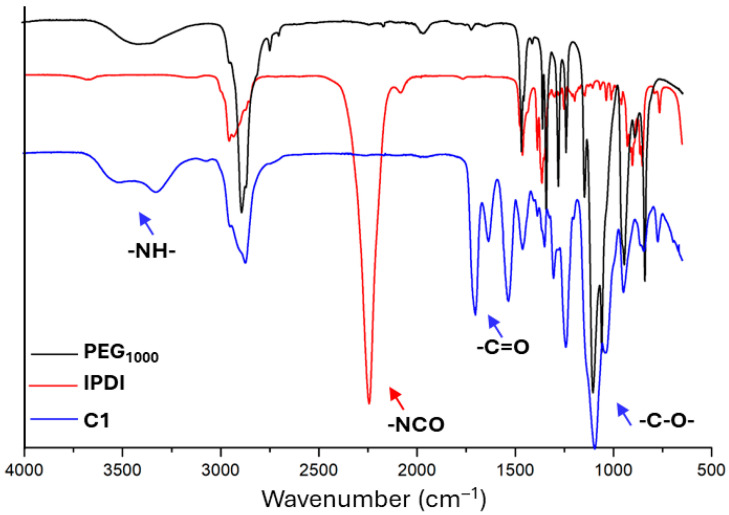
FT-IR spectra of PEG_1000_ (black line), IPDI (red line), and their polymer (sample C1, blue line).

Figure 4 shows the representative FT-IR spectra of the individual components: PEG_1000_ (black line), isophorone diisocyanate (IPDI) monomer (red line), and their corresponding polymer (Sample C1).

The PEG_1000_ IR spectrum shows a typical stretching band of OH groups at 3300–3400 cm^−1^ and of C-H bonds at 2700–2800 cm^−1^, C-O stretching at 1250 cm^−1^, and C-O-C of ether at 1100–1200 cm^−1^. The most characteristic band in the diisocyanate spectra is related to -NCO groups at 2250 cm^−1^. In the polymer spectrum (blue line), the disappearance of -NCO absorbance band, related to diisocyanate monomers, and the appearance of the characteristic band of the -NH bond at 3300–3400 cm^−1^ and C=O stretching around 1700 cm^−1^ confirm the formation of urethane bonds.

### 3.3. Effect of the Composition on Thermoresponsive Behavior

As reported, thermoresponsive polymers exhibit a solution transition at a specific temperature, which causes a sudden change in the solvation state. Polymers become insoluble upon heating and exhibit a so-called lower critical solution temperature (LCST). Below the LCST, the polymer is solvated in water, corresponding to a random coil structure of the polymer chain. However, near the LCST, intermolecular interactions become favored, and the polymer chains change their conformation to a dense globular structure [49]. As reported, the transition temperatures do not represent absolute LCST values but are dependent on concentration. Therefore, these temperatures are more accurately referred to as “cloud point temperatures” (T_CP_), which correspond to the temperature at which PU aggregates begin to form [30,53,54]. The solutions go from transparent to turbid. This effect is well known in thermoresponsive polymers: at higher concentrations, the closer proximity of chains facilitates intermolecular interactions and aggregation, leading to a decrease in the apparent transition temperature. At lower concentrations, polymer chains are more hydrated and dispersed, delaying aggregation and shifting the T_CP_ to higher values [54].

In this study, the T_CP_ was analyzed using UV–Vis spectroscopy by measuring the optical transmittance of polymer solutions (concentration of 1.0 mg mL^−1^) in PBS at various temperatures. Transmittance was monitored at 600 nm as the temperature increased from 10 °C to 60 °C. The cloud point temperature (T_CP_) was defined as the temperature at which transmittance starts to decrease. The T_CP_ values obtained for all the samples are reported in Table 1. As observed, the samples exhibited a critical temperature range of 26 to 49 °C, depending on their composition. In particular, samples C3, C4, C14, and C15 are characterized by T_CP_ values very close to body temperature, making these polymers promising for applications where temperature-induced gelation, such as physiological temperature, is required. In addition, samples characterized by the presence of Hex-DEA functional diol, with a 6-carbon atom alkyl chain on the nitrogen of diethanolamine, are insoluble in buffer solution.

#### 3.3.1. Effect of PEG Molecular Weight

**Figure 5:**
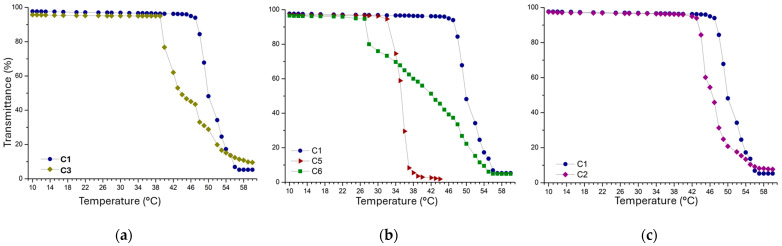
Turbidity curves for polyurethanes: (**a**) C1 and C3 with different PEG molecular weights; (**b**) C1, C5, and C6 with different functional diols; and (**c**) C1 and C2 with different diol:PEG ratios.

The thermoresponsive behavior of PEG-based polyurethanes is primarily determined by the balance between hydrophilic and hydrophobic segments in their molecular structure. In particular, the hydrophilic segment, represented by PEG as the main component, plays a key role [55]. As shown in Figure 4, we compare samples C1 (PEG_1000_-DEA-IPDI) and C3 (PEG_400_-DEA-IPDI), which have the same composition but different molecular weights of PEG: Mw 1000 for sample C1 and Mw 400 for sample C3 (Figure 5a). As the molar mass of the PEG increased from 400 to 1000, the T_CP_ temperature increased from 40.0 to 48.0 °C. This behavior reflects the fundamental role of PEG as the hydrophilic segment of the polyurethane. Longer PEG chains (PEG_1000_) confer higher hydrophilicity and greater chain flexibility, increasing the amount of bound water and delaying dehydration, which results in a higher T_CP_. In contrast, shorter PEG chains (PEG_400_) reduce hydrophilicity and solubility, lowering the T_CP_. Thus, the PEG block acts as the main thermoresponsive component, and its molecular weight is a critical factor in determining transition temperature. The same trend was observed for different PEG-based polymer systems [56,57].

#### 3.3.2. Effect of Functional Diols

To study the effect of functional diols on thermoresponsive behavior, we synthesized diol derivatives from the alkylation of diethanolamine with varying alkyl chain lengths. Graph (b) in Figure 5 displays a comparison of three samples, C1 (PEG_1000_-DEA-IPDI), C5 (PEG_1000_-MeDEA-IPDI), and C6 (PEG_1000_-MeButDEA-IPDI), which have the same compositions but different functional diols: DEA for sample C1, Me-DEA for sample C5, and MeBut-DEA for sample C6. Samples containing Hex-DEA were not characterized due to their insolubility in water. The three samples exhibited markedly different T_CP_ values: 48.0 °C for C1, 33.0 °C for C5, and 28.0 °C for C6. T_CP_ decreased in the order DEA > Me-DEA > MeBut-DEA. This trend can be rationalized based on diol solubility and hydrophobicity. DEA exhibited the highest T_CP_ in PBS due to its free amino group, which enhances solubility and hydrogen bonding with water. The T_CP_ of the Me-DEA-containing sample was higher than that of MeBut-DEA. The longer and bulkier methyl-butyl chain in MeBut-DEA increases hydrophobicity and steric hindrance, further lowering T_CP_ compared to Me-DEA. These results are consistent with literature reports showing that alkylation of amino groups in thermoresponsive polymers reduces solubility and lowers the critical temperature [30,58,59].

#### 3.3.3. Effect of Diol:PEG Ratio

Lastly, we evaluated the effect of the diol:PEG ratio on the polymer’s thermoresponsiveness. Figure 5c compares two samples, C1 and C2, characterized by different diol:PEG ratios: 50:50 for sample C1 and 75:25 for sample C2. This comparison also confirms the influence of the ratio between the hydrophilic and hydrophobic segments on thermoresponsive properties. An increase in the diol functional groups in sample C2 enhances the system’s hydrophobicity and decreases the T_CP_ temperature.

### 3.4. Hydrogel Formation and Sol-Gel Transition

The obtained polymers’ hydrogels were prepared in a PBS solution with a final polymer concentration of 20 wt%. Briefly, the required amount of solid polymer was weighed and dissolved at 4.0 °C overnight in the necessary volume of physiological solution. As observed in the UV–Vis optical transmittance analysis, samples containing Hex-DEA as a functional diol were not soluble in aqueous solution. A tube-inverting test was performed to estimate the gelation temperature of the developed sol-gel systems, referred to as the lower critical gelation temperature (LCGT), defined as the temperature at which no flow was observed [60]. Samples were subjected to a controlled temperature increase from 4.0 to 60 °C in 2.0 °C increments. The temperature was maintained at each step for 5 min, followed by vial inversion and visual inspection. Table 2 reports the physical state and temperature required to obtain hydrogels for the synthesized polymers.

Interestingly, the LCGT values determined at high concentration (20 wt%) are very close to the T_CP_ values obtained at low concentration (1 mg/mL). This similarity suggests that both phenomena are governed by the same PEG dehydration and hydrophobic aggregation process. At low concentration, this process leads only to turbidity (T_CP_), whereas at high concentration, it results in the formation of a continuous three-dimensional network (LCGT). Thus, concentration primarily affects the cooperativity of the transition rather than its absolute onset temperature.

The results obtained and summarized in the table indicate that only samples containing diethanolamine (DEA) as a functional diol can form stable gels above the critical temperature. This phenomenon can be correlated with the free amine groups on the diol functional group, which facilitate the formation of intermolecular bonds responsible for gelling. The alkyl groups present in the diethanolamine derivatives reduce the ability of the polymer chains to interact and bond, thereby increasing the system’s hydrophobicity. This significantly lowers T_CP_ but hinders gelation, likely due to steric hindrance and reduced hydrogen bonding between polymer chains.

These findings are consistent with the thermodynamic nature of the sol-gel transition, which arises from a delicate balance between chain mobility, interchain interactions, and the enthalpy–entropy interplay governing network formation [61]. In the sol phase, polymer chains are relatively dispersed and hydrated, with low viscosity and high mobility. As temperature increases, enthalpic contributions from physical or chemical interactions between chains surpass the entropic favorability of disordered states, leading to the formation of a three-dimensional network and a sharp increase in viscosity—characteristic of the gel phase.

Unlike similar systems reported in the literature, the present hydrogels do not require additional crosslinkers for their formation. Instead, they self-assemble exclusively through non-covalent interactions, such as hydrogen bonding. Notably, our hydrogels retain their structural integrity even when cooled below their critical temperature, demonstrating enhanced stability. This unique behavior makes them particularly promising for biomedical applications where structural stability and stimuli-responsiveness are crucial.

### 3.5. Evaluation of In Vitro Stability and Degradation

A gravimetric analysis was performed to assess the stability and degradation profile of thermoresponsive polyurethane hydrogels in vitro by monitoring the weight loss of samples in a buffer solution at 37 °C, simulating physiological conditions over time [33,62]. The selected samples for analysis were those capable of forming hydrogels and varied in composition, particularly in terms of PEG molecular weight and diol:PEG ratio (Samples C1–C4). The objective was to examine how composition and monomer content influence degradation behavior and, consequently, the material’s stability over time.

Table 3 shows the weight loss values (mean ± standard deviation) obtained for samples C1–C4 during 4 weeks of incubation.

**Figure 6:**
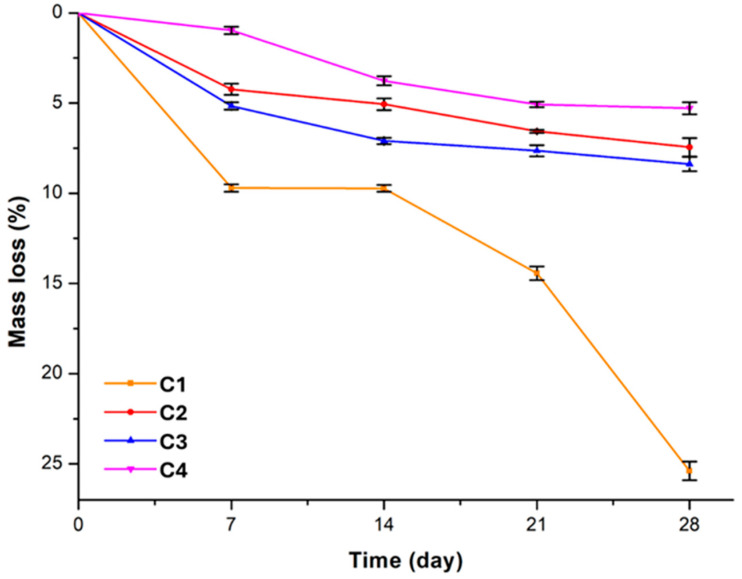
Percentage of mass losses in PBS solutions for up to 28 days at 37 °C for samples C1(PEG_1000_-DEA (50:50) -IPDI), C2 (PEG_1000_-DEA (25:75)-IPDI), C3 (PEG_400_-DEA (50:50)-IPDI), and C4 (PEG_400_-DEA (25:75)-IPDI).

All samples exhibited a weight loss of ~5% to 25% after 4 weeks, confirming overall good in vitro stability, with variations attributed to their composition. In particular, C1 exhibited the lowest in vitro stability, with the highest percentage of degradation after 4 weeks, resulting in a 25.4% mass loss. This sample contains PEG with a molecular weight of 1000, which may have made the system more susceptible to degradation. In contrast, C3, which has the same composition as C1 but with PEG_400_, exhibited greater stability, with a weight loss of only 8.4%. This comparison, shown in Figure 6, highlights the significant influence of PEG molecular weight on degradation behavior, suggesting that shorter polymer chains may contribute to increased resistance to hydrolytic degradation.

The comparison between samples C1 and C2 (Figure 6) as well as C3 and C4 highlights the effect of the diol:PEG ratio on in vitro stability. An increased amount of functional diol, which raises the hydrophobic segment content in the polymer network, leads to reduced degradation. Specifically, C2 shows lower weight loss (7.4%) compared to C1, and C4 (5.3% mass loss) exhibits greater stability than C3, confirming that a higher hydrophobic segment fraction enhances resistance to hydrolytic degradation.

### 3.6. Statistical Analysis

#### 3.6.1. Regression and ANOVA Outcomes

The fitted OLS model achieved an R^2^ of 0.958 and an adjusted R^2^ of 0.917, indicating excellent explanatory power with minimal overfitting. The regression model yielded an adjusted R^2^ indicative of a good fit. PEG molecular weight, type of diol, and, to a lesser extent, the isocyanate and diol:PEG ratio significantly influenced T_CP_. The model equation is as follows:

(3) TCP=41.00+5.75·PEG Mw−13.25·MeDEA−15.75·MeButDEA−0.50·IPDI−1.25·Ratio

Substitution of DEA with Me-DEA or MeBut-DEA reduced T_CP_ by 13.25 °C and 15.75 °C, respectively. An increase in PEG M_w_ from 400 to 1000 resulted in a 5.75 °C increase in T_CP_. The predictive matrix confirmed that formulations with PEG_1000_ and DEA yielded the highest T_CP_ values (~46–47 °C), whereas those with MeBut-DEA and PEG_400_ exhibited the lowest (~23–25 °C). Factorial ANOVA results confirmed the following:

- PEG M_w_ has a statistically significant effect on T_CP_ (*p* = 0.009);
- Diol structure has a highly significant effect on T_CP_ (*p* < 0.001);
- Diisocyanate type and diol:PEG ratio do not significantly affect T_CP_ (*p* = 0.76 and *p* = 0.42, respectively).

#### 3.6.2. Linear Regression Analysis

The ordinary least squares (OLS) regression analysis was conducted to quantitatively assess the influence of four categorical formulation variables on the cloud point temperature (T_CP_). The final model included the PEG molecular weight (PEG M_w_), the type of diol used, the type of diisocyanate, and the molar ratio between PEG and diol. All variables were treated as categorical with reference coding. The regression model exhibited excellent explanatory power, with an R^2^ of 0.958 and an adjusted R^2^ of 0.917. The F-statistic was 22.98, with a corresponding model-level *p*-value of 0.0018, indicating that the overall model was statistically significant. The intercept term (41.0 °C) represents the baseline T_CP_ for a reference formulation composed of PEG_400_, DEA, HMDI, and a 50:50 diol:PEG molar ratio. Each coefficient thus indicates the effect of switching from the reference level to the alternative category.

- PEG molecular weight: The coefficient for PEG_1000_ was +5.75 °C *(p* = 0.009), demonstrating that increasing PEG chain length significantly raises T_CP_. This can be attributed to enhanced hydrophilicity and greater chain mobility.
- Diol structure: The inclusion of alkyl substituents on the diol markedly decreased T_CP_. Specifically, Me-DEA reduced T_CP_ by 13.25 °C (*p* = 0.004) and MeBut-DEA by 15.75 °C (*p* < 0.001) relative to the unsubstituted diol (DEA). These effects reflect the increased hydrophobicity and steric hindrance introduced by the methyl and 3-methylbutyl groups.
- Diisocyanate type: Substitution of HMDI with IPDI had a negligible effect on T_CP_ (–0.50 °C), and the result was not statistically significant (*p* = 0.759). This suggests that the nature of the diisocyanate moiety, under the conditions tested, does not contribute meaningfully to the modulation of thermoresponsiveness.
- Molar ratio: Increasing the diol-to-PEG ratio from 50:50 to 75:25 led to a modest and non-significant decrease in T_CP_ (–1.25 °C, *p* = 0.415), indicating that this parameter plays a minor role compared to the chemical identity of the PEG and diol components.

#### 3.6.3. Factorial ANOVA

**Figure 7:**
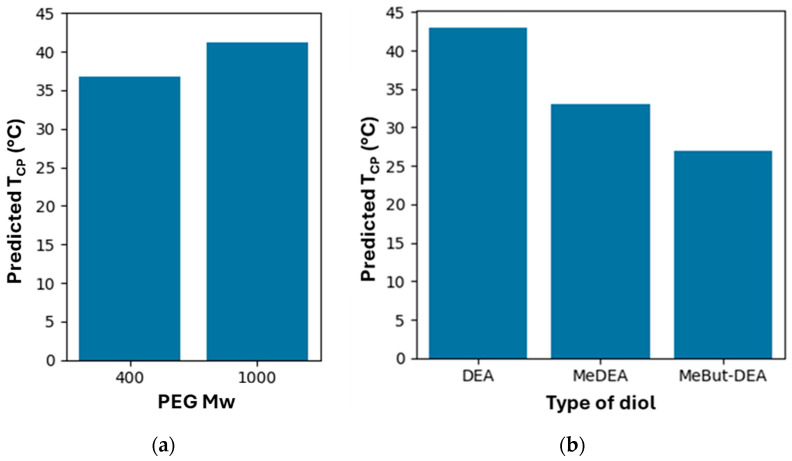
Effect of (**a**) PEG Mw (PEG_400_ and PEG_1000_) and (**b**) type of diol (DEA, Me-DEA, and MeBut-DEA) on predicted T_CP_.

To further confirm the relative importance of each factor, a Type II ANOVA was applied to the fitted regression model. The analysis corroborated the regression findings: both PEG M_w_ and the diol structure were statistically significant sources of variance in T_CP_, with *p*-values of 0.009 and <0.001, respectively. Conversely, the type of diisocyanate and the diol:PEG molar ratio did not significantly affect T_CP_ (*p* = 0.759 and *p* = 0.415, respectively). These results collectively highlight the dominant influence of PEG chain length and diol hydrophobicity on the thermal responsiveness of the copolymer systems studied. The lack of significant contribution from the diisocyanate type implies that the rigid or flexible nature of the linking unit does not disrupt hydration-mediated phase separation under the tested conditions. As shown in Figure 7a, increasing the PEG molecular weight from 400 to 1000 resulted in a higher predicted T_CP_. Figure 7b demonstrates that introducing hydrophobic substitutions on the diol (Me-DEA, MeBut-DEA) significantly decreased the T_CP_, with MeBut-DEA showing the most substantial effect.

The full factorial prediction matrix is reported in Table 4. T_CP_ increases with higher PEG M_w_ and decreases substantially with Me-DEA and MeBut-DEA. The effect of IPDI and a higher diol:PEG ratio (75:25) was minor and not statistically significant.

### 3.7. Predicted Gelation Probability

The logistic regression model successfully estimated the probability of gelation for each experimental formulation. Table 5 summarizes the predicted values. The highest gelation probabilities were observed in samples C13, C14, and C15, which share the combination of DEA as the diol and HMDI as the diisocyanate. These results are consistent with experimental observations. Conversely, the formulations containing Me-DEA or MeBut-DEA showed reduced gelation tendencies, consistent with their lower predicted probabilities and absence of gel formation in experiments.

## 4. Conclusions

This study provides a comprehensive understanding of how molecular design and formulation parameters govern the thermoresponsive behavior and gelation properties of polyurethane-based hydrogels. Through both experimental observations and statistical analysis, it was demonstrated that the chemical composition—specifically, the PEG molecular weight, the type and hydrophobicity of the functional diol, and the diol:PEG ratio—plays a key role in determining the cloud point temperature (T_CP_) and the ability to form stable hydrogels. Regression analysis and factorial ANOVA confirmed that increasing PEG molecular weight enhances hydrophilicity and chain flexibility, leading to higher T_CP_ values. In contrast, the incorporation of alkyl-substituted diols, such as Me-DEA and MeBut-DEA, significantly lowers the T_CP_ by increasing hydrophobic interactions and disrupting hydration, thereby promoting hydrophobic interactions. These results are consistent with the thermodynamic nature of the sol-gel transition, which depends on a balance between chain mobility, intermolecular interactions, and the enthalpy–entropy interplay that governs gelation.

The presence of free amine groups in DEA-based polymers further facilitates intermolecular interactions, contributing to the formation of stable hydrogels above the critical gelation temperature (LCGT). Interestingly, the stability of these hydrogels, even below the LCGT, underscores their potential utility in biomedical applications, such as injectable delivery systems and tissue scaffolding. Their tunable sol-gel transition behavior, directly correlated with the monomer composition, enables precise responsiveness to physiological conditions—a crucial feature for controlled drug release and minimally invasive therapeutic interventions.

Moreover, water plays a dual role in this process, not only as a plasticizing medium that affects viscosity and chain mobility but also as an active participant in network formation via hydrogen bonding and hydration shell reorganization. These solvent-mediated interactions influence both the transition temperature and the kinetic profile of gelation, ultimately affecting the final gel architecture.

The strong correlation observed between T_CP_ (determined via UV–Vis spectroscopy) and LCGT highlights a predictive relationship that can aid in the rational design of thermoresponsive hydrogel systems. Additionally, all formulations tested demonstrated good stability under physiological conditions over 28 days. In particular, samples with higher functional diol content and lower PEG molecular weight exhibited minimal degradation, with mass loss limited to around 5%.

Based on the results mentioned above, it can be concluded that the sol-gel transition in polyurethane-based hydrogels is a finely tunable process governed by both molecular-level design and solvent interactions. These insights provide a solid foundation for the development of advanced, responsive materials tailored for biomedical and soft-matter applications.

## Figures and Tables

**Table 1:** Reagent amounts, ratios for preparing polyurethane polymers, and values of T_CP_.

Samples	PEG M_w_	Diol HO-R’-OH	Diisocyanate OCN-R-NCO	Diol:PEG Molar Ratio	T_CP_ (°C) *
C1	1000	DEA	IPDI	50:50	48 ± 1
C2	1000	DEA	IPDI	75:25	44 ± 1
C3	400	DEA	IPDI	50:50	40 ± 1
C4	400	DEA	IPDI	75:25	39 ± 1
C5	1000	Me-DEA	IPDI	50:50	33 ± 1
C6	1000	MeBut-DEA	IPDI	50:50	28 ± 1
C7	1000	MeBut-DEA	IPDI	75:25	26 ± 1
C8	1000	Hex-DEA	IPDI	50:50	NS
C9	1000	Hex-DEA	IPDI	75:25	NS
C10	400	Hex-DEA	IPDI	50:50	NS
C11	400	Hex-DEA	IPDI	75:25	NS
C12	1000	DEA	HMDI	50:50	49 ± 1
C13	1000	DEA	HMDI	75:25	45 ± 1
C14	400	DEA	HMDI	50:50	40 ± 1
C15	400	DEA	HMDI	75:25	39 ± 1

NS: not soluble. * cloud point temperature, determined from UV–Vis turbidimetry measurements.

**Table 2:** Physical state and critical gelation temperature of the obtained polymers.

Samples	Physical State	LCGT (°C) *
C1	G	48 ± 1
C2	G	45 ± 1
C3	G	41 ± 1
C4	G	40 ± 1
C5	L	-
C6	L	-
C7	L	-
C8	NS	-
C9	NS	-
C10	NS	-
C11	NS	-
C12	G	50 ± 1
C13	G	45 ± 1
C14	G	40 ± 1
C15	G	40 ± 1

G: gel; L: liquid; NS: not soluble. * lower critical gelation temperature estimated by tube-inverting tests.

**Table 3:** Percentage of weight loss (mean ± SD) of the samples over a 4-week incubation period.

Weeks	C1 (PEG_1000_-DEA (50:50) -IPDI)	C2 (PEG_1000_-DEA (25:75)-IPDI)	C3 (PEG_400_-DEA (50:50)-IPDI)	C4 (PEG_400_-DEA (25:75)-IPDI)
1	9.7 ± 0.2	4.2 ± 0.3	5.2 ± 0.2	0.9 ± 0.2
2	9.7 ± 0.2	5.1 ± 0.3	7.1 ± 0.2	3.7 ± 0.2
3	14.4 ± 0.4	6.6 ± 0.1	7.6 ± 0.3	5.1 ± 0.1
4	25.4 ± 0.5	7.4 ± 0.5	8.4 ± 0.4	5.3 ± 0.3

**Table 4:** Predicted T_CP_ (°C) across all formulation combinations.

PEG M_w_	Diol	Diisocyanate	Diol:PEG Ratio	Predicted T_CP_ (°C)
400	DEA	IPDI	50:50	40.50
400	DEA	IPDI	75:25	39.25
400	DEA	HMDI	50:50	41.00
400	DEA	HMDI	75:25	39.75
400	Me-DEA	IPDI	50:50	27.25
400	Me-DEA	IPDI	75:25	26.00
400	Me-DEA	HMDI	50:50	27.75
400	Me-DEA	HMDI	75:25	26.50
400	MeBut-DEA	IPDI	50:50	24.75
400	MeBut-DEA	IPDI	75:25	23.50
400	MeBut-DEA	HMDI	50:50	25.25
400	MeBut-DEA	HMDI	75:25	24.00
1000	DEA	IPDI	50:50	46.25
1000	DEA	IPDI	75:25	45.00
1000	DEA	HMDI	50:50	46.75
1000	DEA	HMDI	75:25	45.50
1000	Me-DEA	IPDI	50:50	33.00
1000	Me-DEA	IPDI	75:25	31.75
1000	Me-DEA	HMDI	50:50	33.50
1000	Me-DEA	HMDI	75:25	32.25
1000	MeBut-DEA	IPDI	50:50	30.50
1000	MeBut-DEA	IPDI	75:25	29.25
1000	MeBut-DEA	HMDI	50:50	31.00
1000	MeBut-DEA	HMDI	75:25	29.75

**Table 5:** Predicted probability of gelation for each sample and experimental observations (physical state).

Sample	Predicted Probability of Gelation	Physical State
C1	0.714	G
C2	0.750	G
C3	0.750	G
C4	0.784	G
C5	0.582	L
C6	0.470	L
C7	0.563	L
C12	0.822	G
C13	0.847	G
C14	0.847	G
C15	0.870	G

G = gel state; L = liquid state.

## Data Availability

The raw data supporting the conclusions of this article will be made available by the authors on request.
